# Circulating resistin levels are early and significantly increased in deceased brain dead organ donors, correlate with inflammatory cytokine response and remain unaffected by steroid treatment

**DOI:** 10.1186/s12967-015-0574-1

**Published:** 2015-06-26

**Authors:** Rille Pullerits, Simona Oltean, Anne Flodén, Mihai Oltean

**Affiliations:** Department of Clinical Immunology and Transfusion Medicine, Sahlgrenska University Hospital, Gothenburg, Sweden; Department of Rheumatology and Inflammation Research, Institute of Medicine, The Sahlgrenska Academy at University of Gothenburg, Gothenburg, Sweden; Organ Donation Unit, Sahlgrenska University Hospital, Gothenburg, Sweden; The Transplant Institute, Sahlgrenska University Hospital, 41345 Gothenburg, Sweden; Department of Surgery, Institute of Clinical Sciences, The Sahlgrenska Academy at University of Gothenburg, Gothenburg, Sweden

**Keywords:** Resistin, Brain death, Organ donor, Cytokines, Steroid treatment

## Abstract

**Introduction:**

Resistin is a pro-inflammatory adipokine that increases after brain injury (trauma, bleeding) and may initiate an inflammatory response. Resistin was found increased in deceased, brain dead organ donors (DBD) and correlated with delayed graft function after kidney transplantation. The kinetics of resistin during brain death (BD), its impact on the inflammatory response and the influence of several donor variables on resistin levels are still unknown.

**Methods:**

Resistin along with a panel of Th1/Th2 cytokines [interferon (IFN)-gamma, interleukin (IL)-1beta, IL-2, IL-6, IL-8, IL10, IL-12, IL-13 and tumor necrosis factor (TNF)] was analyzed in 36 DBDs after the diagnosis of BD and before organ procurement and in 12 living kidney donors (LD). The cytokine levels and resistin were analyzed in relation to donor parameters including cause of death, donors’ age and steroid treatment.

**Results:**

Resistin levels were higher in DBDs both at BD diagnosis and before organ procurement compared to LD (p < 0.001). DBDs had significantly increased IL-1beta, IL-6, IL-8, IL-10 and TNF levels at both time points compared with LD. In DBDs, resistin at BD diagnosis correlated positively with IL-1beta (r_s_ 0.468, p = 0.007), IL-6 (r_s_ 0.511, p = 0.002), IL-10 (r_s_ 0.372, p = 0.028), IL-12 (r_s_ 0.398, p = 0.024), IL-13 (r_s_ 0.397, p = 0.030) and TNF (r_s_ 0.427, p = 0.011) at procurement. The cause of death, age over 60 and steroid treatment during BD did not affect resistin levels. However, steroid treatment significantly decreased pro-inflammatory cytokines IL-1beta, IL-8, TNF and IFN-gamma at the time of organ procurement.

**Conclusions:**

Resistin is increased early in DBDs, remains increased throughout the period of BD and correlates strongly with pro-inflammatory mediators. Resistin level, in contrast to cytokines, is not affected by steroid treatment. Resistin increase is related to the BD but is not influenced by age or cause of death. Resistin may be one of the initial triggers for the systemic inflammatory activation seen in DBDs.

## Background

Deceased brain dead (DBD) organ donors regularly manifest a pronounced inflammatory state resembling the systemic inflammatory response syndrome (SIRS) but its initiating stimuli still remain incompletely known. The increased circulating pro-inflammatory mediators (cytokines, chemokines) released before or during the period between the declaration of brain death and organ procurement lead to an increased apoptosis, inflammatory activation and tissue injury in various organs [1, 2]. These changes increase the susceptibility for both ischemia–reperfusion injury as well as rejection, and may contribute to the inferior results recorded following transplantation of organs from deceased donors as compared with those obtained from healthy, living donors [3, 4].

Resistin, a protein initially described as an adipocyte-secreted peptide in rodents and postulated to contribute to insulin resistance, has recently been shown to play an important role in the various inflammatory conditions [5]. In humans, resistin expression on primary adipocytes is low whereas it is highly expressed on the cells of monocyte-macrophage lineage [5, 6]. Resistin has several features in common with pro-inflammatory cytokines [7, 8]. It promotes inflammation through induction of other cytokines and the expression of resistin itself is strongly up-regulated on the peripheral blood mononuclear cells in response to stimulation with pro-inflammatory cytokines such as IL-6, TNF-α and IL-1β and LPS [7, 9]. In humans, elevated levels of resistin are frequently found in association with autoimmune diseases and inflammation [5]. Resistin has been implicated in obesity, diabetes, and atherosclerosis, cardiovascular and rheumatic diseases [10–13]. Increased resistin levels have also been reported in patients with intracerebral hemorrhage and head trauma [14, 15]. Importantly, we recently showed that increased resistin level measured in brain dead organ donors was associated with delayed graft function and need for dialysis in kidney allograft recipients after transplantation of kidneys from these donors [16]. However, it is unclear whether higher resistin concentrations are present already at the time of diagnosis of brain death and whether resistin further increases during the period between the declaration of BD and organ procurement. Since resistin is able to initiate endothelial activation [17], and induce a pro-inflammatory cytokine response [7], it could commence and further amplify the systemic inflammatory response syndrome and serve as a potential target of the therapy. Therefore, it is imperative to understand the underlying mechanisms and identify therapeutic interventions that may curb the development of the systemic inflammation in the DBD and limit its consequences on the transplantable organs.

Here, we studied the kinetics of resistin in DBD organ donors between the declaration of brain death and organ procurement, the relationship of resistin with cytokines, donor characteristics and pharmacologic interventions in the donor.

## Methods

### Patients and samples

Plasma samples were obtained from 36 DBD organ donors from our procurement area between December 2011 and March 2014. The donors (or next of kin) previously had consented for blood/tissue donation for the purpose of medical research. The study was approved by the Ethical Committee of the University of Gothenburg.

Blood was drawn in ethylene-diamine-tetra-acetic acid (EDTA) containing tubes from DBD donors on the intensive care unit at the time of diagnosis of brain death (acceptance as organ donors) and in the operation room immediately before the start of the organ procurement procedure. The blood was stored at 4°C until centrifugation. Following centrifugation at 2000 rpm for 5 min plasma was recovered, aliquoted and stored at −80°C until analysis. Donor information regarding age, gender and cause of death were retrieved. Methylprednisolone was given to all donors considered for thoracic organ procurement soon after the declaration of brain death and acceptance into donorship (15 mg/kg).

Blood samples were also obtained from healthy individuals donating a kidney for transplantation at our unit (i.e. living donors, LD) the day before surgery. All LD were informed and consented for the sampling and the samples were prepared as described above.

### Measurement of resistin and cytokines

Resistin was measured using a colorimetric sandwich ELISA kit (RnD Systems, Minneapolis, MN, USA) following manufacturer’s instructions. The lower detection limit was 0.16 ng/ml for resistin. The samples also were analyzed for a panel of pro-inflammatory (Th1) and anti-inflammatory (Th2) cytokines. Plasma concentrations of IFN-γ, IL-1β, IL-2, IL-4, IL-6, IL-8, IL-10, IL-12p70, IL-13 and TNF were determined by the electro-chemiluminescence multiplex system Sector 2400 imager from Meso Scale Discovery (Gaithersburg, MD, USA).

### Statistical analyses

Non-parametric statistical methods were used since all continuous variables from DBD as tested for Gaussian distribution with D’Agostino and Pearson omnibus normality test showed non-normal distribution. Kruskal–Wallis test followed by Dunn’s multiple comparisons correction test was used to compare the mean rank values if three or more groups were analysed. Statistically significant relationships were further analyzed using the Mann–Whitney U test. The Wilcoxon signed rank test for paired samples was used to analyze the changes in variables between two time points. Associations between variables were assessed using the Spearman’s rank correlation test and coefficient expressed as Spearman’s rho. Analyses were performed using GraphPad Prism v. 6 (GraphPad software). Data are expressed as median and range, unless otherwise stated. A *p* value <0.05 was considered significant.

## Results

### Donor characteristics

Samples were obtained from 36 DBD donors and 12 living donors. DBD donors were significantly older than LD [median age 64 (range 7–79) vs 46 (34–66), respectively]. Table 1 summarizes the main donor characteristics.

**Table 1:** Donor characteristics (n = 36)

Deceased donors	
Male/Female	20/16
Age (median, range)	64 (7–79)
Cause of death (%)	
Cerebrovascular accident	18 (50%)
Anoxia	12 (33%)
Trauma	6 (17%)
Steroid treatment (%)	21 (58%)
Time between samplings (min)	352 (120–1,200)
Living donors (n = 12)	
Male/Female	3/9
Age (median, range)	46 (34–66)

### Resistin levels in the deceased brain dead and living donors

**Figure 1:**
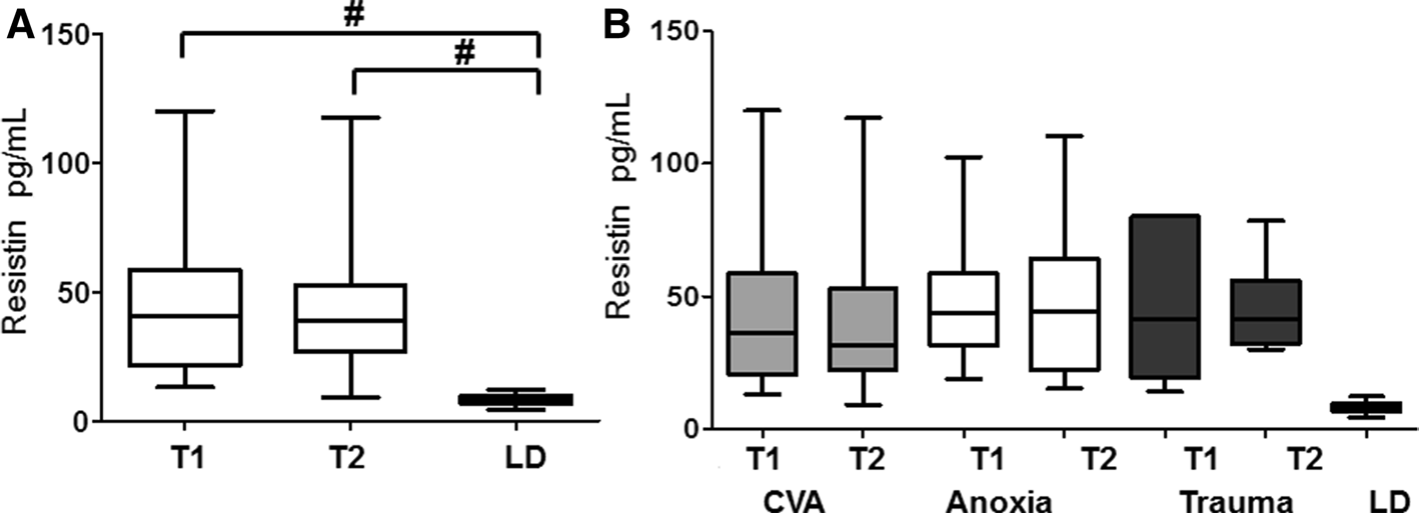
**a** Resistin levels in all the DBD donors at the time of the diagnosis of brain death (T1), immediately before organ procurement (T2) and in living kidney donors (LD). **b** Resistin levels at both timepoints in DBD donors divided according to the cause of death. The *box plots* show medians and interquartile range, *whiskers* show 5–95th percentiles; ^#^p < 0.001; *CVA* cerebrovascular accident.

Median resistin concentration in the LD group was 8 ng/ml (range 4–12 ng/ml). In DBD donors, resistin was found at 4–5-fold higher concentrations than in LD at both time-points (p < 0.0001). Median resistin concentration at the time of diagnosis of BD and acceptance to the donorship was 41 (13–120) ng/ml, while its median concentration immediately before procurement was 39 (9–117) (Figure 1a). No significant differences were observed regarding resistin levels when the DBD donors were stratified according to the cause of brain death (Figure 1b). At the diagnosis of BD, the median resistin concentrations in donors with cerebrovascular bleeding, anoxia and trauma were 36 (13–120) ng/ml, 44 (19–102) ng/ml and 41 (14–80) ng/ml, respectively.

### The cytokine levels in the deceased brain dead and living donors

Serum concentrations of IL-6 and IL-10 were significantly higher in DBD donors compared to LD at the time of brain death diagnosis [IL-6: 152 (4–2,513) ng/ml vs 0.6 (0.3–5.3) ng/ml, p = 0.0001; IL-10: 2.4 (0.3–46.6) ng/ml vs 0.2 (0.1–0.4) ng/ml, p = 0.004, respectively]. At the organ procurement, IL-6 and IL-10 levels in DBD were 91 (3–2,760) ng/ml and 1.8 (0.3–31) ng/ml, respectively. The level of IL-2, IL-8, IL-12, IL-13, and TNF were significantly higher in DBDs at the time of brain death diagnosis as compared to LD.

**Figure 2:**
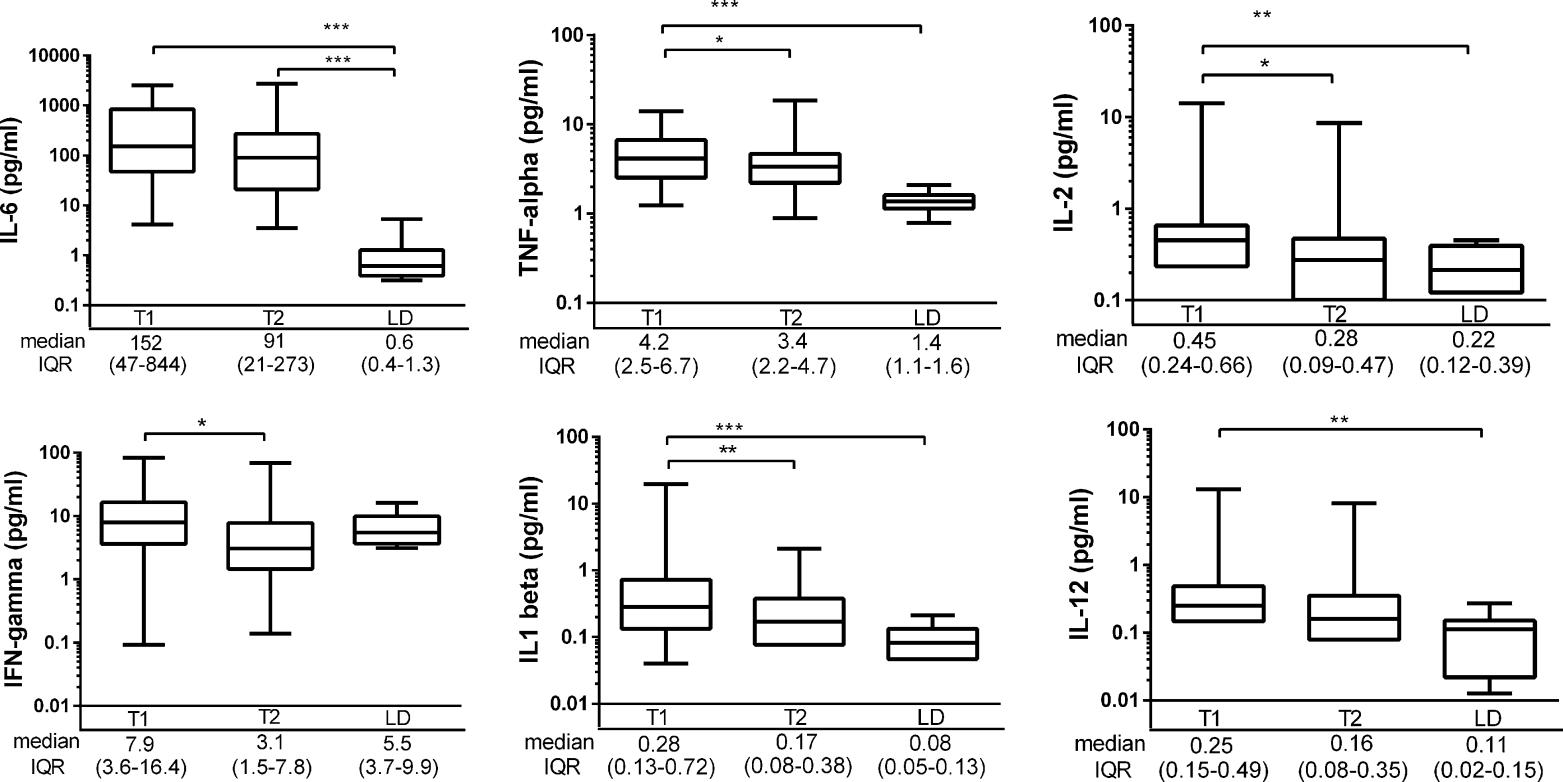
The Th1 cytokines in all the brain dead donors at the time of the diagnosis of brain death (T1) and immediately before organ procurement (T2) and in living kidney donors (LD). The *box plots* on logarithmic scale show medians and interquartile range (IQR), *whiskers* show 2.5–97.5th percentiles. *p < 0.05; **p < 0.01, ***p < 0.001.

**Figure 3:**
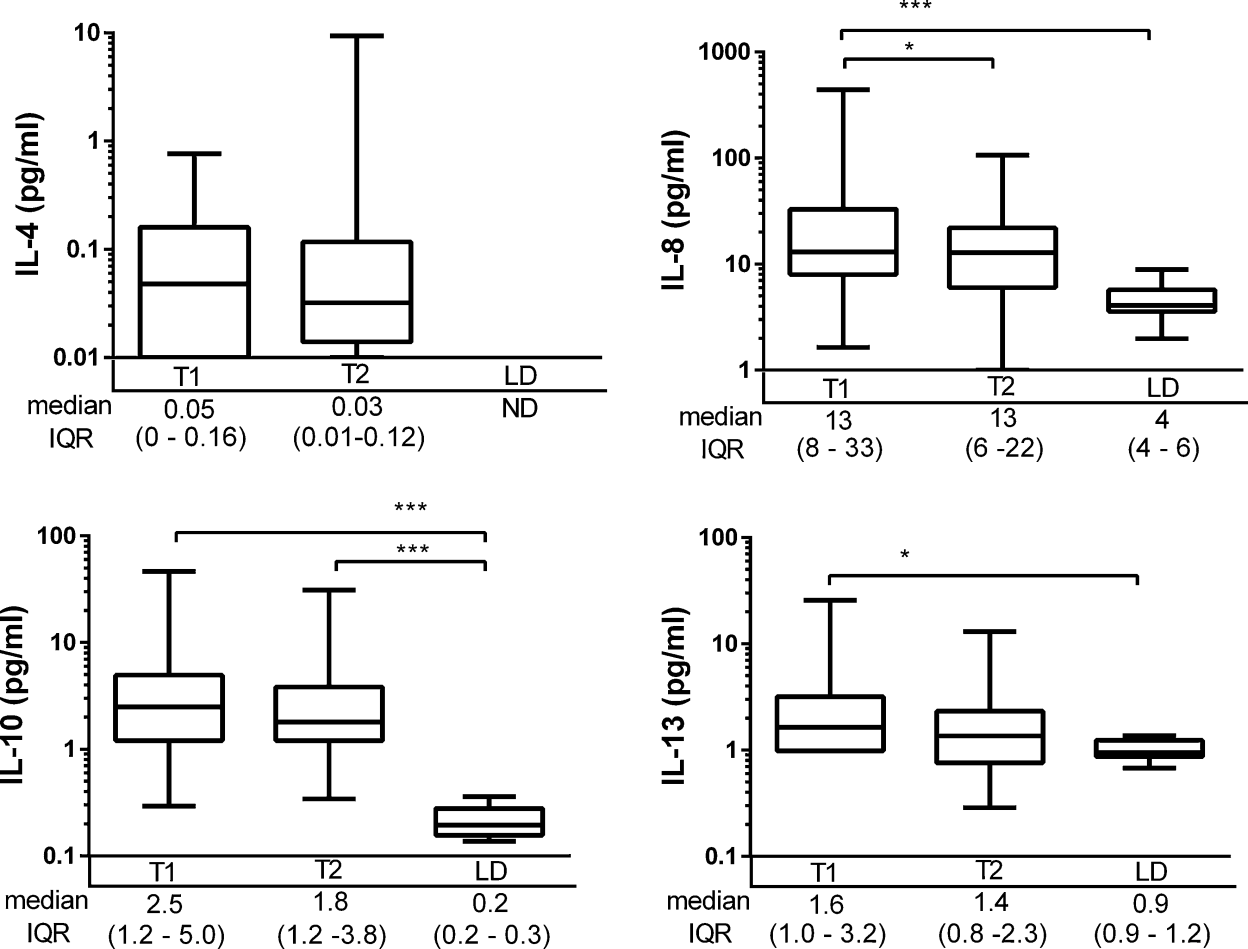
The Th2 cytokines in all the brain dead donors at the time of the diagnosis of brain death (T1) and immediately before organ procurement (T2) and in living kidney donors (LD). The *box plots* on logarithmic scale show medians and interquartile range (IQR), *whiskers* show 2.5–97.5th percentiles. *p < 0.05; ***p < 0.001.

During the period of brain death, IFN-γ, IL-1β, IL-2, IL-8 and TNF significantly decreased and lower values were detected immediately before the organ procurement (T2) compared with the values at the declaration of BD (T1). The concentration of the various Th1 and Th2 cytokines are summarized in Figures 2 and 3.

Overall, the cytokine levels were elevated more than 2 standard deviations above the mean level of that detected in LD in the majority of DBD. IFN-γ was elevated in 29%, IL-2 in 36%, IL-12 in 47%, IL-13 in 56%, IL-1β in 60%, IL-8 74%, TNF in 86%, IL-10 in 94% and IL-6 in 100% of DBD donors. At organ procurement, 97% of DBD donors had high IL-6 and IL-10 levels in relation to LD, and elevated TNF was seen in 77% (Figures 2, 3).

### The relationship between resistin and cytokines

At the time of BD diagnosis, a positive association was found between resistin and IL-1β, IL-2, IL-6, IL-12 and IL-13 levels. Resistin levels measured at the BD diagnosis correlated strongly with IL-6 (r_s_ 0.511, p = 0.002), TNF (r_s_ 0.427, p = 0.011), IL-1β (r_s_ 0.468, p = 0.007) and moderately with IL-10 (r_s_ 0.372, p = 0.028), IL-12 (r_s_ 0.398, p = 0.024), and IL-13 (r_s_ 0.397, p = 0.03) at the time of procurement (Table 2).

**Table 2:** Resistin level at the diagnosis of brain death is associated with cytokines at the diagnosis of brain death (T1) and at organ procurement (T2)

Cytokine	T1 all donors (n = 36)		T2 all donors (n = 36)		T2 untreated donors (n = 12)	
	r_s_	P value	r_s_	P value	r_s_	p value
IFN-gamma	0.034	0.847	0.207	0.234	0.500	0.117
IL-1 beta	0.431	0.010	0.468	0.007	0.673	0.023
IL-2	0.350	0.046	0.248	0.178	0.700	0.016
IL-4	0.129	0.505	0.318	0.114	0.667	0.049
IL-6	0.398	0.018	0.511	0.002	0.727	0.011
IL-8	0.314	0.066	0.116	0.509	0.082	0.81
IL-10	0.217	0.210	0.372	0.028	0.318	0.340
IL-12	0.444	0.009	0.398	0.024	0.697	0.025
IL-13	0.503	0.003	0.397	0.030	0.482	0.133
TNF	0.249	0.149	0.427	0.011	0.455	0.160

The associations were assessed with two-tailed Spearman’s rank correlation test and the correlation coefficient (r_s_) and p value are shown.

### The effect of steroid treatment on resistin and cytokine levels

**Figure 4:**
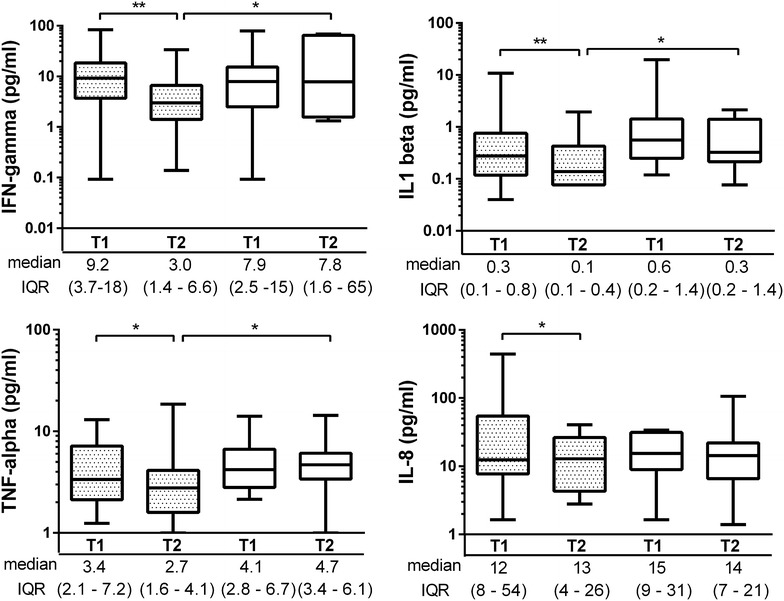
The effect of steroid treatment of the brain dead donors on several cytokines at the time of the diagnosis of brain death (T1) and immediately before organ procurement (T2). The *box plots* show medians and interquartile range (IQR), whiskers show 2.5–97.5th percentiles. *Grey boxes* denote treated DBD donors whereas *open boxes* represent untreated DBD donors. *p < 0.05; **p < 0.01, ***p < 0.001.

**Figure 5:**
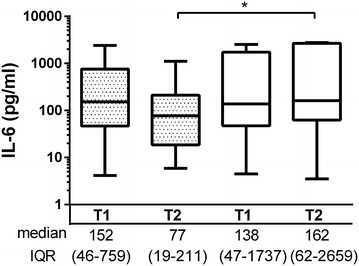
The effect of steroid treatment of the brain dead donors on interleukin-6 at the time of the diagnosis of brain death (T1) and immediately before organ procurement (T2). The *box plots* on logarithmic scale show medians and interquartile range (IQR), *whiskers* show 2.5–97.5th percentiles. *Grey boxes* denote treated DBD donors whereas *open boxes* represent untreated DBD donors. *p < 0.05; **p < 0.01, ***p < 0.001.

The median resistin levels did not differ between DBD donors receiving methylprednisolone treatment (n = 24) or non-treated DBD donors (n = 12) at any time-point. However, methylprednisolone administration during the period between the diagnosis of brain death and organ procurement resulted in lower concentrations of donor IFN-γ, IL-1β, IL-8 and TNF at the time of organ procurement as compared to the levels at diagnosis. Of note, the levels of IFN-γ, TNF and IL-1β were significantly lower in steroid treated BDB donors as compared to the levels in the non-treated DBD donors at the time of organ procurement (Figure 4). Also, IL-6 concentrations were significantly lower in steroid treated DBD donors at the time of organ procurement (p < 0.05) and a trend towards the decrease of IL-6 levels between two time points was seen following steroid treatment (p = 0.09) (Figure 5).

Importantly, the resistin concentration at BD diagnosis correlated strongly with IL-1β (r_s_ 0.672, p = 0.023), IL-2 (r_s_ 0.700 p = 0.016), IL-6 (r_s_ 0.726, p = 0.011) and IL-12 (r_s_, 0.697, p = 0.025) at organ procurement in the DBD donors that did not receive steroid treatment.

Older donors (>60) did not differ at any of the two time points from the younger donors with respect to resistin levels and the studied cytokines.

## Discussion

We recently reported increased resistin at the time of organ procurement but it has been unclear whether this increase occurred in the time elapsed between BD diagnosis and organ donation or this increase was apparent at an earlier moment. The current results indicate an increased plasma resistin and a robust cytokine response already very early in the process of organ donation.

Increased resistin concentration has been detected after the head trauma or intracerebral bleeding and it has been suggested that its concentration is proportional with the magnitude of injury [15, 18]. This increase is quite rapid and significant since resistin increase may occur in head trauma setting within 6 h [14]. We found similar concentrations of resistin as those reported earlier after severe head trauma or intracerebral bleeding. However, during a median observation period range of 2–20 h during the BD we did not observe any further increase suggesting that resistin increase is not a phenomenon specific for the state of brain death but rather of the brain injury leading to the BD, independently of the mechanism of injury.

Accumulating evidence supports a role of human resistin as a pro-inflammatory molecule. Human resistin is highly expressed, up-regulated and secreted from peripheral blood mononuclear cells (PBMC), macrophages and neutrophils in response to various stimuli [6, 9, 19], and in turn, acts on these cells and leads to enhanced inflammatory reaction through activation of intracellular signaling [20]. Although the receptor for resistin is still disputed [21, 22] it is shown that the subsequent intracellular signaling occurs mainly through the NF-κB pathway, the same pro-inflammatory pathway involved in the synthesis and secretion of numerous pro-inflammatory cytokines shifting cells towards an inflammatory phenotype [17, 20, 23]. Resistin has been shown to up-regulate the expression of TNF, IL-6, IL-12 in PBMC and macrophages [7, 20]. In line with these findings, we identified strong positive correlations between the resistin levels at the diagnosis of brain death and several major pro-inflammatory cytokines at both the first and the second time-point during the brain death. The correlation between resistin and TNF, IL-1β and IL-6 were stronger at the latter time-point before organ procurement.

The current results may infer a causal relationship between resistin and these cytokines and indirectly suggest resistin as an initial stimulus for inflammatory status of DBDs but more work at molecular level on clinical samples is needed in order to establish its significance and biological effects. In this setting rodent models of BD may have a limited value as the biologic roles of resistin differ between rodents and humans and rodent models tend to have a shorter duration and more limited possibilities for therapeutic interventions.

The induction of a cytokine response during BD and the inflammatory activation of various organs have earlier been reported both in experimental and clinical setting [1, 24]. This systemic inflammatory response ultimately results in an inflammatory cascade including complement activation, endothelial activation and increased pro-inflammatory mediators. All these molecular events cause direct injury to the various transplantable organs, increase the susceptibility of the future graft to ischemia–reperfusion injury and may ultimately relate with increased recipient mortality after transplantation [25]. Steroid treatment has been shown to abate the levels of several pro-inflammatory factors both in the tissue and in the circulation and may improve the outcome of transplantation [26, 27]. In line with these studies, we found lower levels of pro-inflammatory cytokines at the latter time-point in the DBDs treated with steroids. This suggests that in spite of high levels of resistin still present in circulation, part of its pro-inflammatory effects may be alleviated by steroids.

The present report is from a single-center and has the characteristic limitations of any small series in which the low number and the heterogeneity of the donors prevented further in-depth subgroup analyses and could have made some results prone to a type 2 error. The lack of information on the inflammatory response in the tissues of the various transplantable organs somewhat restricts the interpretation of the data. According to our centers’ policy, biopsies are taken only after graft reperfusion and hence, no tissues from earlier time-points were available for further analyses.

A significant strength is the collection of data from two time-points, allowing for certain mechanistic studies. Moreover, we avoided the confounding influence of the surgical stress on the cytokine response by performing the last sampling before the start of the organ procurement procedure [28]. Although the DBDs studied herein were not consecutive and included only donors who previously consented for the research purposes, the studied group mirrors well the overall donor characteristics at our center with regard to age, gender and cause of death. Our single center study also has the advantage of managing both organ donors and transplant recipients according to similar protocols and routines throughout the study.

## Conclusion

Our results indicate that high resistin levels were detected already early in DBD in the process of organ donation. Resistin levels remained increased throughout the period of brain death and correlated strongly with several major pro-inflammatory circulating mediators. Resistin level, in contrast to cytokines, was not affected by steroid treatment. Our observations propose resistin as an early potential stimulus for the systemic inflammatory response seen during brain death and confirm the ability of methylprednisolone treatment to down-regulate key pro-inflammatory cytokines in DBDs.

### Key messages

- Deceased brain dead donors have greatly increased circulating resistin already at the time of diagnosis of brain death.
- Early resistin levels correlate strongly with several Th1 cytokines immediately before organ procurement.
- Donor treatment with steroids alleviate the cytokine response in spite of high resistin levels.

## Abbreviations

BD: brain death
COD: cause of death
DBD: deceased brain dead donor
ECD: expanded criteria donor
EDTA: ethylenediaminetetraacetic acid
IL: interleukin
IFN: interferon
LD: living donor
